# Effect of testosterone treatment on bone remodelling markers and mineral density in obese dieting men in a randomized clinical trial

**DOI:** 10.1038/s41598-018-27481-3

**Published:** 2018-06-14

**Authors:** Mark Ng Tang Fui, Rudolf Hoermann, Brendan Nolan, Michelle Clarke, Jeffrey D. Zajac, Mathis Grossmann

**Affiliations:** 1 Department of Medicine Austin Health, University of Melbourne, Heidelberg, Australia; 2 Department of Endocrinology, Austin Health, Heidelberg, Australia

## Abstract

To assess the effect of testosterone treatment on bone remodelling and density in dieting obese men, 100 obese men aged 53 years (interquartile range 47–60) with a total testosterone level <12 nmol/L receiving 10 weeks of a very low energy diet (VLED) followed by 46 weeks of weight maintenance were randomly assigned at baseline to 56 weeks of intramuscular testosterone undecanoate (n = 49, cases) or matching placebo (n = 51, controls). Pre-specified outcomes were between-group differences (mean adjusted difference, MAD) in serum c-telopeptide (CTx), N-terminal propeptide of type 1 procollagen (P1NP) and bone mineral density (BMD). At trial end, CTx was significantly reduced in men receiving testosterone compared to placebo, MAD −66 ng/L (95% CI −113, −18), p = 0.018, and this was apparent already after the 10 week VLED phase, MAD −63 ng/L (95% CI −108, −18), p = 0.018. P1NP was marginally increased after VLED, MAD +4.2 ug/L (95% CI −0.01, +8.4), p = 0.05 but lower at study end, MAD −5.6 ug/L (95% CI −10.1, −1.1), p = 0.03. No significant changes in sclerostin, lumbar spine BMD or femoral BMD were seen. We conclude that in obese men with low testosterone levels undergoing weight loss, bone remodelling markers are modulated in a way that may have favourable effects on bone mass.

## Introduction

In men, obesity is commonly associated with lowering of circulating testosterone levels through reduced sex hormone binding globulin and suppression of gonadotropins by visceral adiposity, overriding the age-related increase in SHBG^1^. Such men may be at increased risk of osteoporosis and fracture due to low testosterone levels^2^ and possibly, abdominal obesity^3^. Estradiol is the major regulator of bone in men, and the effects of testosterone on bone are considered to be predominantly indirect, with low testosterone being a risk factor for osteoporosis largely because less substrate is available for conversion to estradiol^4,5^. In addition, low testosterone is associated with low muscle mass, and sarcopenic obesity has been associated with increased falls and fracture risk in older men^6^.

Diet and exercise is the initial best approach to assist with weight loss in obese men. Weight loss confers a multitude of health benefits but may have adverse effects on bone health. Marked weight loss after bariatric surgery is associated with substantial reductions in bone mineral density (BMD)^7^. Lesser degrees of weight loss through diet are associated with more modest reductions in BMD, occurring predominantly at the hip^8^. Diet-associated bone loss has been demonstrated even in the context of an exercise program, suggesting that exercise may not be sufficient to prevent bone loss during caloric restriction^9^. The bone loss is mediated through increased bone remodelling, and proposed mechanisms include mechanical unloading due to diet-associated loss of muscle, the primary source of bone-anabolic stimuli, and possibly reduced secretion of anabolic myokines or adipokines^10^.

In randomised controlled clinical trials, testosterone treatment leads to improvements in areal and volumetric BMD at the spine and hip^11–14^. Testosterone modulates bone remodelling either directly or via aromatisation to estradiol by activating sex steroid receptors in bone cells^15^. In addition, testosterone increases muscle mass which may have indirect anabolic effects on bone mass^16^.

We have recently shown that in dieting obese men with lowered testosterone levels, testosterone treatment prevented the diet-associated loss of muscle mass^17^, and improved androgen deficiency-related symptoms^18^. However, whether concomitant testosterone treatment in dieting obese men has effects on bone is not known. In this secondary, but pre-specified analysis of a previously described randomised controlled clinical trial among dieting obese men with low testosterone^17^, we tested the hypothesis that testosterone treatment mitigates detrimental effects of weight loss on bone health.

## Material and Methods

### Design Overview

The study design has been reported in detail previously^17^. Briefly, this study was a randomized, double-blind, placebo-controlled trial (RCT) (ClinicalTrials.gov NCT01616732, registered June 8, 2012) of intramuscular testosterone undecanoate treatment or matching placebo in oily base, administered for 56 weeks, in men following a strict diet and exercise weight loss program. The diet consisted of eight weeks of very low energy diet (VLED) providing approximately 640 kcal, 1,190 mg of calcium and 8.2 μg (328 IU) of vitamin D daily, which was weaned over two weeks and from weeks 10–56 subjects were instructed to follow an energy restricted diet based on the Australian Commonwealth Scientific and Industrial Research Organisation Total Wellbeing diet providing 1,350 kcal/day. Subjects were instructed to undertake 30 minutes of moderate- intensity exercise daily. The trial was conducted from April 2013 through to November 2015 at a tertiary referral centre (Austin Health, Melbourne, Australia). The trial protocol was approved by the local ethics committee (Austin Health Human Research Ethics Committee 2012/04495) and each participant provided written informed consent. All research was performed in accordance with relevant guidelines/regulations.

We followed the CONSORT checklist of information to include when reporting a randomised trial (Supplementary Table 1).

### Setting and Participants

Obese (BMI >30 kg/m^2^) community-dwelling men with two fasting, morning (8–10 am) total testosterone (TT) levels ≤12 nmol/L, confirmed on two separate occasions, were eligible to participate in the trial. The exclusion criteria were outlined previously^17^ and included organic hypogonadism due to pituitary or testicular disease, contraindications to testosterone treatment, use of weight-altering medications including insulin, previous VLED failure and bariatric surgery. Baseline testosterone and estradiol were measured by electrochemiluminescence immunoassay (ECLIA) (Roche Diagnostics, North Ryde NSW, Australia) as used at the study hospital for routine clinical care and were remeasured by liquid chromatography-tandem mass spectroscopy (LCMS/MS) at study end from stored samples frozen at −80 °C^19^. Intra-assay CVs for the testosterone ECLIA were 2.8% at 7.4 nmol/L and 3.5% at 45.0 nmol/L, and inter-assay CVs were 4.0% at 7.4 nmol/L and 4.8% and 31 nmol/L. Intra-assays CV for the estradiol ECLIA were 8.4% at 69 pmol/L and 1.8% at 700 pmol/L, and inter-assay CVs were 5.0% at 283 pmol/L and 3.0% at 1,500 pmol/L. For the testosterone LCMS/MS, within-day CVs were 2.7% at 2.7 nmol/L and 5.3% at 31.3 nmol/L, and between-day CVs were 9.0% at 2.8 nmol/L and 10.1% at 38.0 nmol/L. For the estradiol LCMS/MS, within-day CVs were 13.0% at 35.2 pmol/L and 11.1% at 132.4 pmol/L, and between-day CVs were 14.5% at 37 pmol/L and 11.0% at 152 pmol/L (Professor David J. Handelsman, ANZAC Research Institute, Concord, NSW, Australia, *personal communication*).

Free testosterone was calculated (calculated free testosterone (cFT)) according to Vermeulen^20^, using measured SHBG and albumin concentrations. SHBG was measured by electrochemiluminescence immunoassay (Roche Cobas C8000), with intra- and inter-assay CVs <2.0% and of 3.4% at 44 nmol/L, respectively. Albumin was measured by bromocresol purple (BCP) assay (Roche Cobas 8000), with intra- and inter-assay CVs 1.5% and of 1.4% at 41 g/L, respectively.

Subjects were randomly assigned in a concealed 1:1 allocation to either testosterone or placebo using a block of size four with equal probability to the two treatments within four strata accounting for BMI (≤ or >37 kg/m^2^) and age (≤ or >60 years)^17^. The randomization sequence was generated by an independent statistician and implemented by the Austin Health clinical trials pharmacists. Participants, trial investigators and pharmacists were blinded to treatment allocation. Participants received either 1000 mg testosterone undecanoate (the standard ampoule strength in Australia) or visually identical placebo in oily base by deep intramuscular buttock injection at weeks 0, 6 (manufacturer-recommended loading dose), 16, 26, 36 and 46 to ensure therapeutic trough levels of 10–15 nmol/L. In addition, all subjects received a VLED providing 640 kcal per day for 10 weeks followed by a 46 week weight maintenance diet based on the Australian Commonwealth Scientific and Industrial Research Organisation (CSIRO) Total Wellbeing diet as described^17^.

### Main Outcome Measures

The primary outcome of the RCT was the change in fat mass^17^. The main pre-specified outcome measures of this secondary analysis were the changes in serum c-telopeptide (CTx) and in N-terminal propeptide of type 1 procollagen (P1NP), both measured on a Roche Cobas e602 platform (Roche Diagnostic International Ltd, Rotkreuz, Switzerland) with inter-assay CVs of 2.5–3.9%. The reference ranges for CTx were 400–900 ng/L (males aged 20–24 years), 100–600 ng/L (males aged 25–70 years), for P1NP 15–115 μg/L (males aged 20–24 years), 15–80 μg/L (males aged 25–70 years). As an additional end-point, sclerostin was assayed on an R&D systems ELISA kit (R&D systems Inc. Minneapolis, MN, USA) with inter-assay CV of 4.5%, and a reference range of 31.3–1,000 pg/mL. All study samples were stored at −80°C followed by assay batch testing of all samples for each bone remodelling marker in a single assay run at study end. Areal bone mineral density (BMD) at the lumbar spine, femoral neck and total femur were measured using the dual energy X-ray absorptiometry (DEXA) Lunar Prodigy system (GE Healthcare Australia, Parramatta, NSW 2150, Australia) with CVs of 1.1–2.6% based on three repeated scans in 10 subjects within 3 weeks aged 22–46 years old. All scans were performed on the same machine supervised by the same technician. Quality control was performed using the Lunar quality assurance calibration block as supplied by and per manufacturer’s recommendations and additionally by daily scanning of an anthropomorphic spine phantom. T scores were derived from male reference ranges using enCore software v13.6 provided by the manufacturer. Physical activity levels were measured using the GT3x accelerometer (ActiGraph, Pensacola, FL, USA).

### Schedule of Assessment and Measurements

Blood tests, bone density and physical activity levels were measured at weeks 0 (before any intervention), 10 and 56. Additional study visits at weeks 2, 4, 6, 16, 26, 36 and 46 were undertaken for clinical assessment, administration of injections and to ensure compliance with the prescribed diet. An independent safety investigator reviewed week 26 safety parameters for pre-defined withdrawal criteria: haemoglobin >180 g/L, hematocrit >0.54 or PSA >5.5 μg/L.

### Statistical Methods

Comparison of baseline characteristics was based on Welch’s t-test for normally distributed parameters, as assessed by the Kolmogorov-Smirnov test with Lilliefors correction or Wilcoxon rank-sum test in case of non-normal distribution. Data shown are mean (standard deviation) or median [IQR]. Correlations are based on Kendall’s Tau rank test.

Changes in CTx, P1NP, sclerostin, bone density and physical activity levels between cases and controls over the follow-up visits were analysed using a linear mixed model with restricted maximum likelihood estimator. The model included random intercepts per subject and, as fixed variable, the baseline levels of the respective outcome variable, three categorical time points (at 0, 10 and 56 weeks), the group (testosterone and placebo), and the interaction of time point x group. The latter represents the measure of interest (between-group change over time), which was quantitatively expressed as mean adjusted difference (MAD) surrounded by the profiled 95% confidence interval (CI). P values for the change from 0 to 10 and from 0 to 56 weeks were conventionally adjusted for multiple testing by the Holm-Bonferroni method. A sensitivity analysis was carried out under the non-ignorable missingness hypothesis using multilevel joint modelling multiple imputation^21,22^. This accounts for missing data uncertainty by producing imputations from a range of plausible regression parameters. We used 100 imputed data sets to obtain pooled estimates, which were retained with a spread of 20,000 for each after 100,000 burn-in-iterations.

All tests were two-tailed with p < 0.05 denoting statistical significance. No adjustments were made for multiple comparisons on other variables. Analyses were conducted using R version 3.3.3 for Mac^23^ with the lme4 package version 1.1–12^24^ jomo 2.5-1^21^ and mitml 0.3–5^22^ and SPSS version 22 (IBM, Armonk, NY, USA).

### Data availability

The datasets generated during and/or analysed during the current study are available from the corresponding author on reasonable request

## Results

### Study Subjects

Of 584 men who expressed an interest in participating in the trial, 264 men proceeded to screening investigations and 164 were ineligible, chiefly due to a TT level >12 nmol/L (N = 158)^17^. The remaining 100 men were randomized to testosterone (49, cases) or placebo (51, controls). 82 men completed the trial, 44/49 (90%) cases and 38/51 (75%) controls (p = 0.099). The most common reason for non-completion was failure to attend visits (testosterone = 3, placebo = 12)^17^. All men underwent baseline BMD at the lumbar spine, femoral neck and total femur except for one man who had bilateral hip replacements and did not have a femoral neck bone density performed. As use of anti-resorptive agents was not an exclusion criterion to participating in the original trial, for this sub-study, one man (receiving testosterone) was excluded from the analyses in this study due to the use of risedronate prior to and during the trial. The use of calcium and vitamin D supplements was infrequent and both its use and serum levels were balanced between the randomised groups and did not change during the study. Only one man (placebo) was using calcium supplements. 13 men receiving placebo and 11 receiving testosterone were using vitamin D supplements. No participant was commenced on any anti-resorptive agent, calcium or vitamin D supplements during the study (nor were these altered) during the trial and no man sustained a fracture during the course of the trial.

Baseline characteristics were largely comparable between the groups (Table 1). Median BMD T-scores were normal at the lumbar spine, femoral neck and total femur, and median vitamin D level was not deficient.

**Table 1:** Baseline characteristics of randomly assigned study participants.

	Testosterone group (n = 48)	Placebo group (n = 51)	P value
Age (y)	54.2 [46.9, 59.3]	52.8 [47.4, 60.1]	0.95
Weight (kg)	118 (15.8)	121 (19.6)	0.54
BMI (kg/m^2^)	37.4 [34.8, 40.6]	37.3 [34.7, 41.6]	0.51
TT LCMS/MS(nmol/L)	6.8 (2.0)	7.0 (1.6)	0.86
TT ECLIA (nmol/L)	8.5 [6.60, 10.2]	8.4 [7.10, 9.95]	0.75
cFT LCMS/MS (pmol/L)	159 (46)	172 (44)	0.16
cFT ECLIA (pmol/L)	200 [162, 240]	202 [172, 238]	0.40
SHBG (nmol/L)	25 [18, 31]	21 [17, 26]	0.21
E2 LCMS/MS (pmol/L)	122 (73)	128 (58)	0.69
E2 ECLIA (pmol/L)	66.0 [50.0, 87.5]	86 [66.0, 107]	0.006
Lumbar spine T-score	0.1 [−0.8, 0.9]	0.2 [−0.4, 1.5]	0.25
Lumbar spine BMD (g/cm^2^)	1.22 [1.14, 1.33]	1.24 [1.18, 1.38]	0.30
Femoral neck T-score	−0.1 [−0.8, 0.6]	0.1 [−0.8, 0.9]	0.50
Femoral neck BMD (g/cm^2^)	1.05 [0.96, 1.15]	1.08 [0.97, 1.18]	0.38
Total femur T-score	0.4 [−0.3, 0.8]	0.6 [−0.3, 1.3]	0.20
Total femur BMD (g/cm^2^)	1.16 [1.06, 1.22]	1.19 [1.05, 1.29]	0.22
Vitamin D (nmol/L)	49 [33, 58]	52 [33, 63]	0.69
CTx (ng/L)	314 [240, 382]	306 [222, 376]	0.29
P1NP (ug/L)	43.5 [35.8, 47.5]	37.0 [29.5, 49.0]	0.07
Sclerostin (pg/mL)	148 [121, 205]	172 [123, 232]	0.26
Steps per day	6378 [4761, 7543]	6371 (4816, 7440)	0.79
Activity (%/day)	14.2 (5.7)	13.5 (4.5)	0.79

Data are median [IQR], or mean (SD), based on normality testing, using the Kolmogorov-Smirnov test with Lilliefors correction. P values were calculated for the difference between groups using the Wilcoxon rank-sum test, or Welch’s t-test. P < 0.05 was considered significant.

TT, total testosterone by LCMS/MS; cFT, calculated free testosterone; SHBG, sex hormone binding globulin; E2, estradiol; BMD, bone mineral density.

Among men aged ≥50 years, 18 were osteopenic (lowest T score at any site −1.0 to −2.5) and none were osteoporotic (T score <−2.5). Among men aged <50 years, two had a Z-score <−2.0. We performed a separate subgroup analysis was performed on these 20 men who may be considered to have low BMD (see sensitivity analyses below). Four men had CTx levels above the upper limit of the age-related reference range and one man had a P1NP level above the upper limit of the age-related reference range. By study end, trough TT increased to 14.1 nmol/L (recommended trough range 10–15 nmol/L) in cases and to 10.0 nmol/L in controls, both p < 0.05 compared to baseline and significantly different between groups (p < 0.001)^17^. The on-study testosterone and estradiol treatment levels are provided in Supplementary Table 2.

### Change in Main Outcome Measures

**Figure 1:**
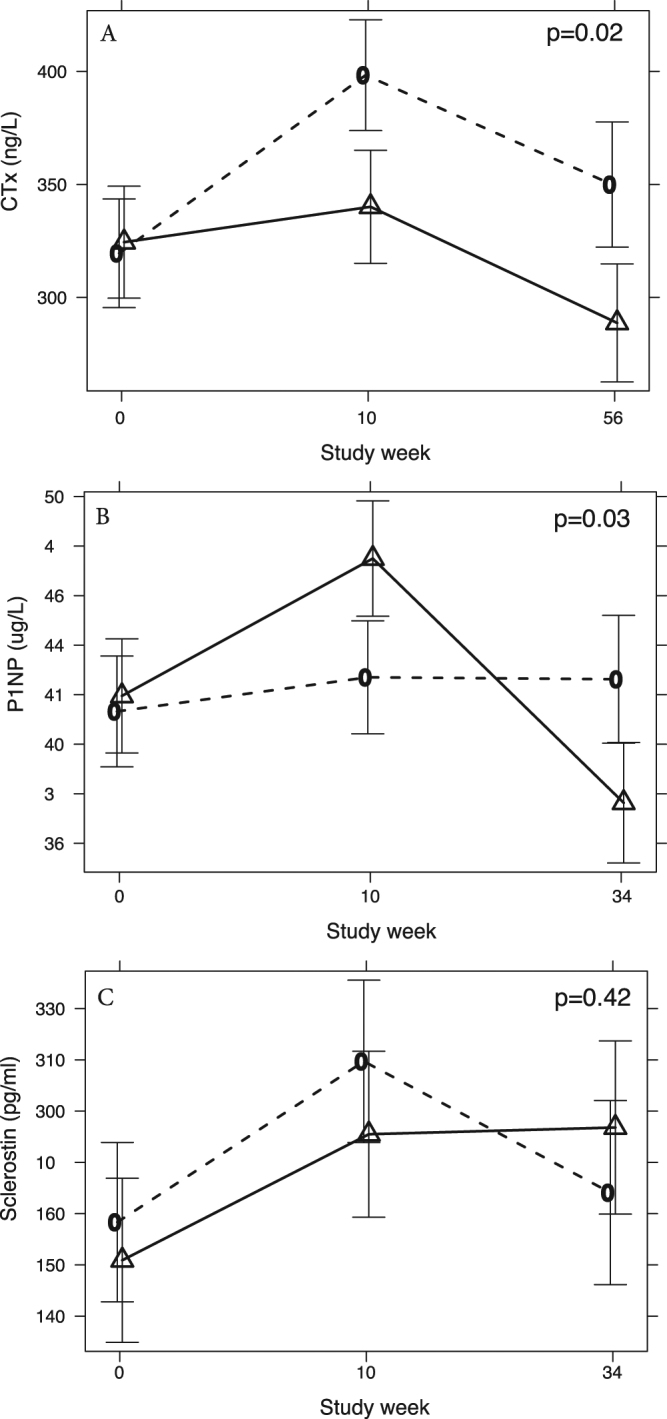
Shown are adjusted mean (95% CI) circulating CTx (**A**), P1NP (**B**) and sclerostin (**C**) levels in testosterone- (solid lines) and placebo- (dashed lines) treated men at baseline (week 0), after the 10 week VLED phase (week 10), and after the subsequent 46 week maintenance phase (week 56, study end) of the trial.

**Figure 2:**
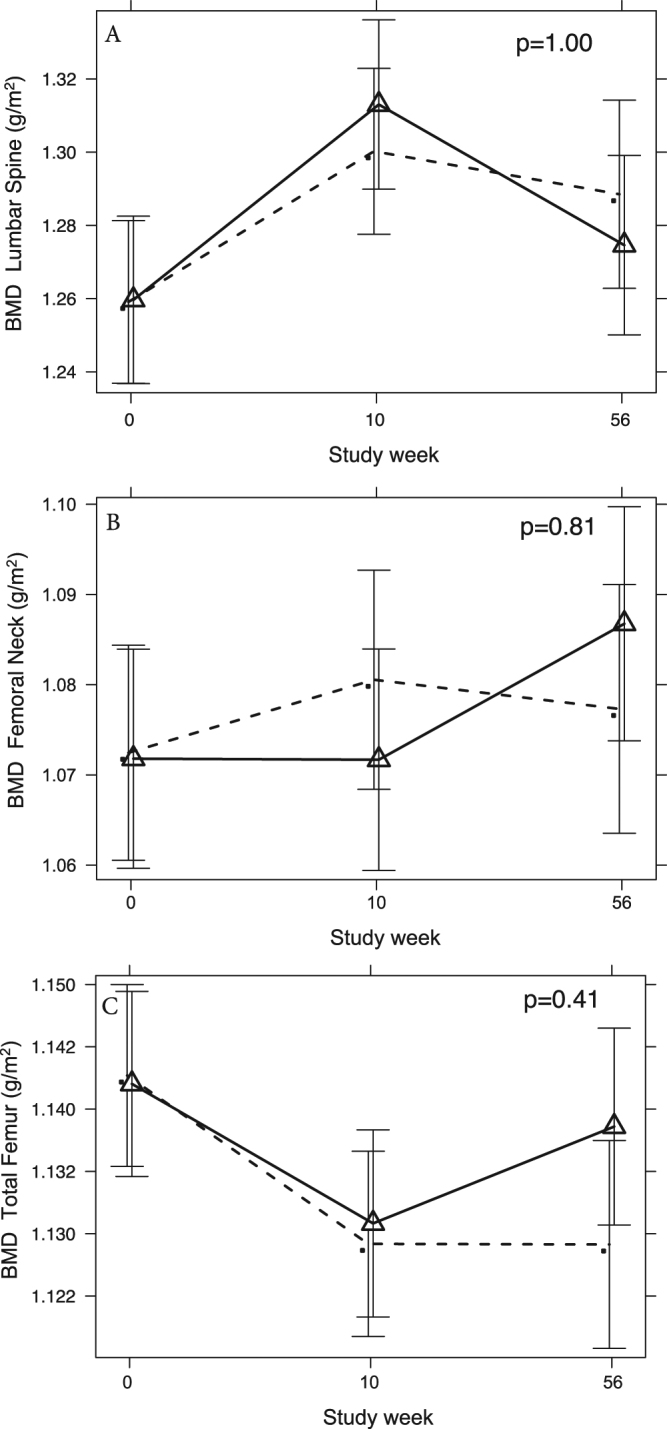
Shown are adjusted mean (95% CI) BMD at the lumbar spine (**A**), femoral neck (**B**) and total femur (**C**) in testosterone- (solid lines) and placebo- (dashed lines) treated men at baseline (week 0), after the 10 week VLED phase (week 10), and after the subsequent 46 week maintenance phase (week 56, study end) of the trial.

Using a linear mixed model including adjustment for baseline differences in the outcome of interest and incorporating data points week 0, 10 and 56, CTx was significantly reduced in dieting men receiving testosterone treatment compared to placebo at trial end, MAD −66 ng/L (95% CI −113, −18), p = 0.018 **(**Fig. 1A, Table 2**)**. Similarly, P1NP was significantly reduced in dieting men receiving testosterone treatment compared to placebo at trial end, MAD −5.6 ug/L (95% CI −10.1, −1.1), p = 0.03 **(**Fig. 1B, Table 2**)**. There was no difference in sclerostin levels between groups, MAD −20 pg/mL (95% CI −11, 51), p = 0.42 (Fig. 1C, Table 2). The effect of testosterone to lower CTx compared to placebo was apparent as early as week 10 of the trial (the end of the rapid weight loss VLED phase), MAD −63 ng/L (95% CI −108, −18), p = 0.018. In contrast, during this period, testosterone lead to a marginal increase in P1NP compared to placebo MAD +4.2 ug/L (95% CI −0.01, +8.4), p = 0.05. There was no difference in BMD at either the lumbar spine (MAD −0.01, 95% CI −0.06; 0.03, p = 1.0), femoral neck (MAD 0.01, 95% CI −0.01; 0.03, p = 0.81) and total femur (MAD 0.01, 95% CI 0.00; 0.02, p = 0.41) **(**Fig. 2A–C, Table 2**)** following 56 weeks of testosterone treatment. Baseline BMD at the femoral neck was weakly correlated with baseline estradiol levels (0.15, p = 0.03), total fat mass (0.27, p < 0.0001) and lean mass (0.20, p = 0.003). No such correlations between BMD at the lumbar spine or total femur and sex hormones or body composition parameters, existed. No significant negative correlation of visceral fat and BMD at the lumbar spine or femur were apparent. Moreover, there was no correlation between the changes in serum testosterone or serum estradiol on treatment with either bone remodelling markers or BMD parameters.

**Table 2:** Main study outcomes.

	Testosterone	Testosterone	Placebo	Placebo	MAD^a^	P
	Week 0 (n = 48)	Week 56 (n = 44)	Week 0 (n = 51)	Week 56 (n = 38)		
CTx (ng/L)	314 [240, 382]	256 [200, 336]	306 [222, 376]	337 [253, 407]	−66 [−113, −18]	0.02
P1NP (ug/L)	43.5 [35.8, 47.5]	36.0 [28.5, 44.0]	37.0 [29.5, 49.0]	41.0 [35.2, 48.0]	−5.6 [−10.1, −1.1]	0.03
Sclerostin (pg/mL)	148 [121 205]	168 [121, 257]	172 [123, 232]	191 [146, 246]	20 [−11, 51]	0.42
Lumbar spine T-score	0.05 [−0.75, 0.92]	0.25 [−0.50, 1.30]	0.20 [−0.35, 1.45]	0.40 [−0.08, 1.48]	0.12 [−0.09, 0.33]	0.53
Lumbar spine BMD (g/cm^2^)	1.22 [1.14, 1.33]	1.25 [1.13, 1.38]	1.24 [1.18, 1.38]	1.26 [1.21, 1.40]	−0.01 [−0.06, 0.03]	1.00
Femoral neck T-score	−0.05 [−0.80, 0.60]	−0.10 [−0.80, 0.78]	0.10 [−0.80, 0.88]	0.05 [−0.90, 0.95]	0.01 [−0.18, 0.20]	0.92
Femoral neck BMD (g/cm^2^)	1.05 [0.96, 1.15]	1.06 [0.97, 1.16]	1.08 [0.97, 1.18]	1.08 [0.95, 1.19]	0.01 [−0.01, 0.03]	0.81
Total femur T-score	0.40 [−0.28, 0.80]	0.30 [−0.55, 0.95]	0.60 [−0.30, 1.30]	0.60 [−0.20, 1.10]	0.06 [−0.04, 0.15]	0.73
Total femur BMD (g/cm^2^)	1.16 [1.06, 1.22]	1.15 [1.03, 1.24]	1.19 [1.05, 1.29]	1.19 [1.07, 1.26]	0.01 [0.00, 0.02]	0.41

Data are median [IQR] for testosterone and placebo groups.

^a^Mean adjusted difference (MAD) refers to the between- group change at week 0 (commencement of the RCT) and week 56.

### Sensitivity analyses

Using multilevel joint modelling multiple imputation (see Methods), main outcomes of the ITT analysis with the incomplete data were confirmed, CTx MAD at week 56–60 ng/L (95% CI −114, −6), p = 0.03. The loss of efficiency, fraction of missing information at trial end was 0.29, and the relative increase in sample variability attributed to the missing data was 0.40. Subject variability explained 17% of total variation. For P1NP, MAD was −3.8 µg/L (95% CI −8.4, 0.8), p = 0.11 at week 56. Fraction of missing information was 0.12, relative variability increase 0.13 and intra class correlation 0.14.

In a subgroup analysis by interaction, patients with low BMD at the beginning of the trial (n = 20, 9 in the testosterone group and 11 the placebo group, p = 0.92) did not significantly differ in their outcomes, CTx (MAD −48 ng/L (95% CI −107, 11), p = 0.33) and P1NP (MAD −2.0 (95% CI −7.7, 3.7), p = 0.92 at week 56), compared to the “normal” group (n = 79). Likewise, there were no significant differences in outcomes when baseline femoral neck T-score was used as a continuous variable (CTx: MAD 12 ng/L (95% CI −10, 34), p = 0.58, and P1NP: MAD −0.41 ng/L (95% CI −2.6, 1.7), p = 0.71).

### Adverse Events

As reported previously^17^, there was no difference in overall adverse events, or serious adverse events which were few, between the two groups. Three men were withdrawn from the study; one man due to a PSA rise (testosterone group) and two men because of a major cardiovascular event (one each in testosterone and placebo group).

## Discussion

In this secondary analysis of a RCT among obese men with low testosterone levels subjected to a rigorous weight loss program, we found that testosterone modulated bone remodelling markers in a direction that may be expected to increase in bone mass. However, there were no apparent differences in BMD between the testosterone and placebo treated men.

As previously reported^17^, testosterone and placebo treated men lost similar amounts of body weight (12 to 13.5 kg) during the initial 10-week VLED phase, and this weight loss was maintained in both groups at the end of the 56-week RCT. Interestingly, compared to placebo, the bone formation marker P1NP was slightly (p = 0.05) higher in testosterone-treated men at the end of the VLED phase, but lower (p = 0.03) by study end. In contrast, the bone resorption marker CTx was lower in testosterone treated men throughout the study. In a 6-month RCT of testosterone treatment among hypogonadal men^25^, Wang *et al*. similarly reported an initial but non-sustained increase in the bone formation marker procollagen, and sustained lowering of urinary N-telopeptide, a marker of bone resorption. However, men were not subjected to weight loss in that study^25^. Thus, even during caloric restriction, testosterone treatment may lead to a temporary increase in bone formation and a sustained reduction on bone resorption. However, in our RCT, these differences in bone remodelling markers were not associated with significant between group differences in BMD, either at the lumbar spine or the femoral neck. While our RCT was not designed to detect a difference in BMD between testosterone and placebo treated men, our study had 80% power at p-level of 0.05 to detect an effect size of 0.58 standard deviations in BMD, corresponding to 0.08 g/cm^2^ in lumbar spine BMD. Given the importance of estradiol in the control of bone remodelling and bone mineral density in men^4,5^, an additional explanation for the non-significant effect of testosterone treatment on BMD may be the fact that baseline circulating estradiol concentrations in our cohort were not particularly low with LCMS/MS medians in the testosterone and placebo group of 122 and 128 pmol/L respectively, above LCMS/MS thresholds reported to increase rates of bone loss in two different cohorts of older community-dwelling men of 114 pmol/L^4,5^ and 98 pmol/L^25^ respectively. Previous 12-month RCTs of older men with low testosterone have reported modest, but significant increases of BMD ranging from 1.2% to 3.5% at the lumbar spine^12,26^, so our study should not be too short to demonstrate an effect. However, one 36-month RCT reported an 8.9% increase in lumbar spine BMD^13^, suggesting that longer treatment may be associated with a more robust response. However, in contrast to our study, men were not subjected to a concomitant weight loss program in these previous RCTs.

In our study, we did not find any differences in sclerostin levels between testosterone and placebo treated men. Sclerostin is an inhibitor of the Wnt signalling pathway and circulating levels have been reported to increase with diet induced weight loss in older obese adults, although this increase is mitigated by exercise^26^. Dihydrotestosterone decreases sclerostin in cultured osteocytes via an androgen receptor mediated pathway, and sclerostin levels were inversely associated with circulating testosterone levels in a small cross-sectional cohort study^27^. However, in an elegant 3-week block-and-replace study designed to selectively withdraw and replace testosterone and estradiol by Khosla’s group, estradiol rather than testosterone prevented increases in sclerostin that occur with induced testosterone and estradiol depletion^28^. Although testosterone treatment led to parallel increases in circulating estradiol levels in our study, we did not see an effect of testosterone treatment on circulating sclerostin levels in the context of weight loss. However, given bone markers were first measured 10 weeks into the trial, we may have missed earlier changes.

The adipokine leptin, a regulator of energy homeostasis has been implicated in the regulation of bone mass, both via direct effects on bone cells as well as via central actions on hypothalamic neurons (for review see^29^). Weight loss leads to reductions in leptin levels, with an associated increase in bone resorption markers^30,31^. The sustained reduction in CTx in testosterone-treated men compared to men receiving placebo is consistent with previous studies reporting reduced leptin resistance following testosterone treatment^32,33^. The strengths of this double-blind placebo controlled RCT include the unique design combining testosterone treatment with a rigorous weight loss program and the focus on obese men with lowered testosterone levels. While men who had pathological androgen deficiency were excluded from this study, 97% and 89% of our participants had baseline free testosterone levels below <243 and <220 pmol, the respective cut-offs reported for healthy young men^34^ and for men with late onset hypogonadism^35^. Moreover, the mean baseline testosterone (6.9 nmol/L) and free testosterone (166 pmol/L) levels in our cohort were substantially lower than the mean levels TT and cFT levels of 13.5 nmol/L and 280 pmol/L reported for unselected, community dwelling obese men^35^. We therefore focussed on a group of men in whom testosterone treatment is not yet considered routine. The administration of testosterone or placebo by study staff resulting in 100% compliance, and the similar weight loss between testosterone and placebo treated men allowing unbiased group comparisons of DEXA measures. We followed the intention to treat principle and used statistical models that are robust against values missing at random, making it unlikely that differential drop out rates led to spurious results.

Limitations include that this is a secondary analysis of a RCT designed to assess the effects of testosterone treatment on fat and lean mass. In contrast to previous RCTs of similar size and duration^36,37^, we did not find any effects of testosterone treatment on bone density. However, these previous RCTs^36,37^ did not incorporate a weight loss program, and enrolled participants who were older than our middle-aged cohort. We cannot exclude the possibility that our RCT was underpowered, although the study had 80% power to detect an effect size of 0.58 standard deviations in BMD, corresponding to 0.08 g/cm^2^ in lumbar spine BMD at a p-level of 0.05. Moreover, the differences in bone remodelling markers observed here, albeit modest are consistent with a favourable effect of testosterone treatment on bone mass, even in the context of weight loss. Alternatively, we cannot exclude the possibility that testosterone is unable to increase BMD in the setting of caloric restriction. Moreover we observed a modest increase in circulating testosterone (+2.9 mmol/L)^17^ in the placebo group consistent with previous weight loss studies^38^, and this may have contributed to the apparent lack of a between group difference in BMD. Finally, men were not selected on the basis of reduced BMD, and interestingly, despite the low baseline testosterone levels, median BMD was normal in this cohort.

In conclusion, among obese men with low-normal testosterone levels subjected to a rigorous weight loss program, men treated with testosterone had reduced bone remodelling compared placebo-treated men. However, this was not associated with higher BMD in testosterone treated men, despite reduction of bone remodelling, preservation of muscle mass^17^ and increased spontaneous activity^17^. Reassuringly, the absence of marked BMD changes between groups suggest that at least in the short term, modest weight loss in obese men with low testosterone may not have detrimental effects on bone health, although studies are necessary to confirm these findings.

## Electronic supplementary material

Dataset 2

## References

[1] Camacho EM (2013). Age-associated changes in hypothalamic-pituitary-testicular function in middle-aged and older men are modified by weight change and lifestyle factors: longitudinal results from the European Male Ageing Study. Eur. J. Endocrinol..

[2] Fink HA (2006). Association of testosterone and estradiol deficiency with osteoporosis and rapid bone loss in older men. J. Clin. Endocrinol. Metab..

[3] Sogaard AJ (2015). Abdominal obesity increases the risk of hip fracture. A population-based study of 43,000 women and men aged 60–79 years followed for 8 years. J. Int. Med..

[4] Khosla S (1998). Relationship of serum sex steroid levels and bone turnover markers with bone mineral density in men and women: a key role for bioavailable estrogen. J. Clin. Endocrinol. Metab..

[5] Barrett-Connor E (2000). Low levels of estradiol are associated with vertebral fractures in older men, but not women: the Rancho Bernardo Study. J. Clin. Endocrinol. Metab..

[6] Scott D (2017). Sarcopenic obesity and its temporal associations with changes in bone mineral density, incident falls, and fractures in older men: The Concord Health and Ageing in Men Project. J. Bone Miner. Res..

[7] Coates PS, Fernstrom JD, Fernstrom MH, Schauer PR, Greenspan SL (2004). Gastric bypass surgery for morbid obesity leads to an increase in bone turnover and a decrease in bone mass. J. Clin. Endocrinol. Metab..

[8] Zibellini J (2015). Does diet-induced weight loss lead to bone loss in overweight or obese adults? A systematic review and meta-analysis of clinical trials. J. Bone Miner. Res..

[9] Schwartz AV (2012). Effect of 1 year of an intentional weight loss intervention on bone mineral density in type 2 diabetes: results from the Look AHEAD randomized trial. J. Bone Miner. Res..

[10] Schafer AL (2016). Decline in Bone Mass During Weight Loss: A Cause for Concern?. J. Bone Miner. Res..

[11] Wang C (2004). Long-term testosterone gel (AndroGel) treatment maintains beneficial effects on sexual function and mood, lean and fat mass, and bone mineral density in hypogonadal men. J. Clin. Endocrinol. Metab..

[12] Snyder PJ (2017). Effect of testosterone treatment on volumetric bone density and strength in older men with low testosterone: a controlled clinical trial. JAMA Int. Med..

[13] Amory JK (2004). Exogenous testosterone or testosterone with finasteride increases bone mineral density in older men with low serum testosterone. J. Clin. Endocrinol. Metab..

[14] Snyder PJ (1999). Effect of testosterone treatment on bone mineral density in men over 65 years of age. J. Clin. Endocrinol. Metab..

[15] Manolagas SC, O’Brien CA, Almeida M (2013). The role of estrogen and androgen receptors in bone health and disease. Nat. Rev. Endocrinol..

[16] Bhasin S (2006). Drug insight: testosterone and selective androgen receptor modulators as anabolic therapies for chronic illness and aging. Nat. Clin. Pract. Endocrinol. Metab..

[17] Ng Tang Fui M (2016). Effects of testosterone treatment on body fat and lean mass in obese men on a hypocaloric diet: a randomised controlled trial. BMC Medicine.

[18] Fui NT, Hoermann M, Prendergast R, Zajac LA, Grossmann JD (2017). M. Symptomatic response to testosterone treatment in dieting obese men with low testosterone levels in a randomized, placebo-controlled clinical trial. Int. J. Obes. (Lond.).

[19] Harwood DT, Handelsman DJ (2009). Development and validation of a sensitive liquid chromatography-tandem mass spectrometry assay to simultaneously measure androgens and estrogens in serum without derivatization. Clin. Chim. Acta.

[20] Vermeulen A, Verdonck L, Kaufman JM (1999). A critical evaluation of simple methods for the estimation of free testosterone in serum. J. Clin. Endocrinol. Metab..

[21] Carpenter, J. R., Kenward, M. G. Multiple imputation and its application in *Statistics and**Clinical Practice* (eds Carpenter, J. R., Kenward, M. G.) 156–197 (Wiley, 2013).

[22] Grund, S., Robitzsch, A. & Lüdtke, O. Mitml: tools for multiple imputation in multilevel modeling (Version 0.3-5). https://cran.r-project.org/package=mitml (2017).

[23] R Core Team. R: A language and environment for statistical computing. R Foundation for Statistical Computing, Vienna, Austria. https://www.R-project.org/ (2017).

[24] Bates K, Machler M, Bolker BM, Walker SC (2015). Fitting linear mixed-effects models using lme4. J. Stat. Softw..

[25] Wang C (2001). Effects of transdermal testosterone gel on bone turnover markers and bone mineral density in hypogonadal men. Clin. Endocrinol. (Ox.f).

[26] Armamento-Villareal R (2012). Weight loss in obese older adults increases serum sclerostin and impairs hip geometry but both are prevented by exercise training. J. Bone. Miner. Res..

[27] Di Nisio A (2015). Regulation of sclerostin production in human male osteocytes by androgens: experimental and clinical rvidence. Endocrinology.

[28] Modder UI (2011). Regulation of circulating sclerostin levels by sex steroids in women and in men. J. Bone Miner. Res..

[29] Dimitri P, Rosen C (2017). The central nervous system and bone metabolism: an evolving story. Calcif. Tissue Int..

[30] Bruno C (2010). Serum markers of bone turnover are increased at six and 18 months after Roux-en-Y bariatric surgery: correlation with the reduction in leptin. J. Clin. Endocrinol. Metab..

[31] Prouteau S, Benhamou L, Courteix D (2006). Relationships between serum leptin and bone markers during stable weight, weight reduction and weight regain in male and female judoists. Eur. J. Endocrinol..

[32] Fui NT, Hoermann M, Grossmann R (2017). M. Effect of testosterone treatment on adipokines and gut hormones in obese men on a hypocaloric diet. J. Endo. Soc..

[33] Jockenhovel F (1997). Testosterone substitution normalizes elevated serum leptin levels in hypogonadal men. J. Clin. Endocrinol. Metab..

[34] Bhasin S (2011). Reference ranges for testosterone in men generated using liquid chromatography tandem mass spectrometry in a community-based sample of healthy nonobese young men in the Framingham Heart Study and applied to three geographically distinct cohorts. J. Clin. Endocrinol. Metab..

[35] Wu FC (2010). Identification of late-onset hypogonadism in middle-aged and elderly men. N. Engl. J. Med..

[36] Kenny AM, Prestwood KM, Gruman CA, Marcello KM, Raisz LG (2001). Effects of transdermal testosterone on bone and muscle in older men with low bioavailable testosterone levels. J. Gerontol. A. Biol. Sci. Med. Sci..

[37] Kenny AM (2010). Effects of transdermal testosterone on bone and muscle in older men with low bioavailable testosterone levels, low bone mass, and physical frailty. J. Am. Geriatr. Soc..

[38] Corona G (2013). Body weight loss reverts obesity-associated hypogonadotropic hypogonadism: a systematic review and meta-analysis. Eur. J. Endocrinol..

