# Supplementary material for: Effect of testosterone treatment on bone remodelling markers and mineral density in obese dieting men in a randomized clinical trial
Source: Sci Rep. 2018 Jun 14;8:9099. doi: 10.1038/s41598-018-27481-3 (PMC6002535; doi:10.1038/s41598-018-27481-3)
Supplement: Supplementary file 1 — Dataset 2 [file 41598_2018_27481_MOESM1_ESM.docx]

**Effect of testosterone treatment on bone remodelling markers and mineral density in obese dieting men in a randomized clinical trial**

Mark Ng Tang Fui, Rudolf Hoermann, Brendan Nolan, Michelle Clarke, Jeffrey D. Zajac, Mathis Grossmann

**Supplementary Table 2. On study testosterone and estradiol levels**

|  | T wk 0  (N=48) | T wk 10  (N=47) | T wk 26  (N=46) | T wk 56  (N=44) | P wk 0  (N=51) | P wk 10  (n=49) | P wk 26  (n=43) | P wk 56  (N=38) | MAD ^a^ | P |
| --- | --- | --- | --- | --- | --- | --- | --- | --- | --- | --- |
| TT ECLIA (nmol/L) | 8.5 [6.6;10.2] | 19.3 [16.3;22.6] | 14.7 [11.1;17.2] | 14.3 [11.9;18.7] | 8.4 [7.1;10.0] | 11.0 [9.5;12.6] | 11.5  [9.4;13.7] | 10.0  [8.5;11.6] | 5.8  [3.7; 7.8] | <0.001 |
| E2 ECLIA (nmol/L) | 66  [50;88] | 143 [121;184] | 109 [88.0;156] | 109 [73.;170] | 86.0 [66.;107] | 88 [65;103] | 78 [57;103] | 76 [50;102] | 61 [43;79] | 0.03 |

Data are median [IQR] for testosterone (T) and placebo (P) groups.

^a^Mean adjusted difference (MAD) refers to the between- group change at week 0 (commencement of the RCT) and week 56.
